# Characterization of antibiotic resistance in commensal bacteria from an aquaculture ecosystem

**DOI:** 10.3389/fmicb.2015.00914

**Published:** 2015-09-08

**Authors:** Ying Huang, Lu Zhang, Laura Tiu, Hua H. Wang

**Affiliations:** ^1^Department of Food Science and Technology, The Ohio State University, Columbus, OHUSA; ^2^South Centers, The Ohio State University, Piketon, OHUSA; ^3^Department of Microbiology, The Ohio State University, Columbus, OHUSA; ^4^School of Biological Sciences, Fudan University, Yangpu, ShanghaiChina

**Keywords:** antibiotic resistance, aquaculture ecosystem, multiple risk factors, commensal bacteria

## Abstract

The objective of the study was to improve the understanding of antibiotic resistance (AR) ecology through characterization of antibiotic-resistant commensal isolates associated with an aquaculture production system. A total of 4767 isolates non-susceptible to sulfamethoxazole/trimethoprim (Sul/Tri), tetracycline (Tet), erythromycin (Erm), or cefotaxime (Ctx), originated from fish, feed, and environmental samples of an aquaculture farm with no known history of antibiotic applications were examined. Close to 80% of the isolates exhibited multi-drug resistance in media containing the corresponding antibiotics, and representative AR genes were detected in various isolates by PCR, with feed isolates had the highest positive rate detected. Identified AR gene carriers involved 18 bacterial genera. Selected AR genes led to acquired resistance in other bacteria by transformation. The AR traits in many isolates were stable in the absence of selective pressure. AR-rich feed and possibly environmental factors may contribute to AR in the aquaculture ecosystem. For minimum inhibitory concentration test, brain heart infusion medium was found more suitable for majority of the bacteria examined than cation-adjusted Mueller Hinton broth, with latter being the recommended medium for clinical isolates by standard protocol. The data indicated a need to update the methodology due to genetic diversity of microbiota for better understanding of the AR ecology.

## Introduction

The rapid emergence of antibiotic resistance (AR) in the global ecosystem has become a major public health concern. In the past decade, with a broadened scope of investigation, both population- and organism-based studies have illustrated that commensal bacteria likely have played a key role in AR ecology (Andremont, 2003; Wang et al., 2006; Wang and Schaffner, 2011). With the expansion of research territory, AR has been found prevalent across the ecosystem, from clinical samples, animal and human hosts, retail foods, to waste water, soil, and other natural environment (Zhanel et al., 2000; Österblad et al., 2001; Aubry-Damon et al., 2004; Wang et al., 2006; Koike et al., 2007; Sommer et al., 2009; Li and Wang, 2010; D’Costa et al., 2011; Zhang, 2011; Ye et al., 2013). For instance, using non-specific culturing methods and culture-independent real-time PCR, the abundance of AR in the food chain was revealed and a broad-spectrum of non-pathogenic and even beneficial bacteria were found carrying transmissible AR genes (Duran and Marshall, 2005; Wang et al., 2006; Manuzon et al., 2007). Characterization of antibiotic-resistant (ART) isolates associated with food animals, ready-to-eat food products, human, and environmental samples has led to the discovery of various mechanisms involved in AR evolution (Sommer et al., 2009; Bhullar et al., 2012), enrichment (Zhang et al., 2011), and persistence (Sørum et al., 2006; Rosvoll et al., 2010; Li et al., 2011). It becomes evident that commensal flora provides a particular window of vulnerability of AR in the ecosystem, and prompt response to the early indication can be critically important for preventive AR management (Andremont, 2003; Wang et al., 2006; Wang and Schaffner, 2011). Due to the advancement in metagenomics and other molecular techniques, the complexity of microbiome associated with both hosts and environment has been gradually uncovered (Venter et al., 2004; Sommer et al., 2009; Zhang et al., 2011). However, the extremely large size and diverse distribution of the commensal population, as well as genetic diversity of the subpopulations presented several major challenges to researchers. There is no single way of recovering, culturing, or characterizing all the cultures, making proper interpretation of data a daunting task.

Aquatic creatures are susceptible to infectious diseases. Use of antibiotics in aquaculture production systems is considered a major risk factor contributing to AR in aquaculture products and the ecosystem. ART pathogens, such as *Aeromonas* (Akinbowale et al., 2007; Penders and Stobberingh, 2008), *Vibrio* (Oh et al., 2011; Rebouças et al., 2011), and *Salmonella* (Ribeiro et al., 2010; Budiati et al., 2013), have been reported to be associated with the aquaculture system. Particularly, certain AR determinants have been found in both aquaculture and clinical isolates, indicating potential AR dissemination between food and humans. For instance, one gene cassette containing *bla*_CMY -2_, *sugE*, and *blc* detected in *Aeromonas salmonicida* ssp. *salmonicida* isolates from Atlantic Canadian salmon farm was identical to a transponson-like element widely distributed among clinical and food-borne *Salmonella* and other Enterobacteriaceae throughout Asia and the United States (McIntosh et al., 2008). Rhodes et al. (2000) reported the dissemination of *tet*(A)-associated Tn*1721* and Tn*1721*-like elements among different *Aeromonas* species and *Escherichia coli*, and between the human and aquaculture environment in distinct geographical locations (including Norway, Scotland, England, and Germany). In addition, various AR genes were found in a broad spectrum of commensal bacteria associated with aquaculture products and the environment (Ye et al., 2013; Shah et al., 2014). However, these data are still insufficient in elucidating AR risk factors in the aquaculture ecosystem.

In a recent study, we have uncovered a rich profile of ART bacteria in samples from a domestic aquaculture farm with no known history of antibiotic application, by culture-dependent and -independent methods (Huang, 2014). The objective of this study was to characterize the genotypic and phenotypic features of the corresponding ART isolates, as well as AR persistence and dissemination, for an improved understanding of the AR ecology associated with aquaculture production. During the evaluation of minimum inhibitory concentration (MIC) for representative antibiotics of the isolates, we have compared the data using both standard approach for pathogens (CLSI, 2013) and a modified method (Wang et al., 2006), to collect baseline information for further methodology improvement, to address the needs for proper assessment of commensal bacteria.

## Materials and Methods

### Bacterial Strains and Culture Condition

A total of 4767 isolates recovered from Sul/Tri-, Tet-, Erm-, or Ctx-containing agar plates were examined in the study. These isolates originated from fish intestine (1045 isolates), fish surface rinsing water (850 isolates), fish feed (150 isolates), pond mud (1293 isolates), and pond water (1429 isolates) from an aquaculture farm with no known history of antibiotic applications. All isolates were cultured in brain heart infusion (BHI) media containing the corresponding antibiotics, including 152 μg/ml of sulfamethoxazole (Sigma–Aldrich, St. Louis, MO, USA) with 8 μg/ml of trimethoprim (Sigma–Aldrich), 16 μg/ml of tetracycline (Sigma–Aldrich), 100 μg/ml of erythromycin (Fisher Scientific, Waltham, MA, USA), or 4 μg/ml of cefotaxime (Sigma–Aldrich; Ye et al., 2013).

### Determination of Drug Resistance Profiles, AR Genes, and Identification of AR Gene Carriers

Recovered Sul/Tri^r^, Tet^r^, Erm^r^, and Ctx^r^ isolates were spotted on BHI agar plates containing each of the four antibiotics for rapid assessment of their phenotypic resistance profile.

Approximately one-fourth of the ART isolates were randomly selected for conventional PCR screening for representative AR genes (Li and Wang, 2010). AR gene primers used in the study were listed in **Table 1**. Approximately 10% of the positive PCR products were confirmed by DNA sequence assessment using an ABI Prism 3700 sequencer (Applied Biosystems, Foster City, CA, USA) at the Plant Microbe Genomics Facility, The Ohio State University, and the obtained DNA sequences were compared with published AR gene sequences deposited in the NCBI database. Approximately 50% of the AR gene carriers were further identified by PCR and partial 16S rRNA gene sequence analysis, as described previously (Wang et al., 2006).

**Table 1:** Primers used for conventional PCR.

Primer	Sequence (5′–3′)	Reference
*sul*1 F	CGGCGTGGGCTACCTGAACG	Kerrn (2002)
*sul*1 R	GCCGATCGCGTGAAGTTCCG	
*sul*2 F	GCAGGCGCGTAAGCTGA	Zhang et al. (2011)
*sul*2 R	GGCTCGTGTGTGCGGATG	
*tet*S F	GAACGCCAGAGAGGTATT	Zhang et al. (2011)
*tet*S R	TACCTCCATTTGGACCTCAC	
*tet*L F	TTGGATCGATAGTAGCC	Zhang et al. (2011)
*tet*L R	GTAACCAGCCAACTAATGAC	
*tet*M F	CGAACAAGAGGAAAGCATAAG	Zhang et al. (2011)
*tet*M R	CAATACAATAGGAGCAAGC	
*erm*B F	TGGTATTCCAAATGCGTAATG	Zhang et al. (2011)
*erm*B R	CTGTGGTATGGCGGGTAAGT	
*erm*C F	GCTAATATTGTTTAAATCGTCAAT	Wang et al. (2006)
*erm*C R	TCAAAACATAATATAGATAAA	
*bla*_TEM_ F	CATTTCCGTGTCGCCCTTATTC	Dallenne et al. (2010)
*bla*_TEM_ R	CGTTCATCCATAGTTGCCTGAC	
*bla*_SHV_ F	AGCCGCTTGAGCAAATTAAAC	Dallenne et al. (2010)
*bla*_SHV_ R	ATCCCGCAGATAAATCACCAC	
*bla*_OXA_ F	GGCACCAGATTCAACTTTCAAG	Dallenne et al. (2010)
*bla*_OXA_ R	GACCCCAAGTTTCCTGTAAGTG	
*bla*_CMY -2_ F	GACAGCCTCTTTCTCCACA	Zhang et al. (2011)
*bla*_CMY -2_ R	TGGAACGAAGGCTACGTA	
*bla*_CTXMU1_	ATGTGCAGYACCAGTAARGT	Zhang et al. (2011)
*bla*_CTXMU2_	TGGGTRAARTARGTSACCAGA	

### MIC Assessments

Twenty-nine ART isolates carrying AR genes were subjected to the MIC test. The assessments were conducted using microbroth dilution method as suggested by CLSI (2013) protocol. BHI and cation-adjusted Mueller Hinton (CAMH) broth were employed as basic medium independently. ART isolates were grown in the broth containing the corresponding antibiotic (including up to 608 μg/ml Sul with 32 μg/ml Tri, 512 μg/ml Tet, 512 μg/ml Erm, or 512 μg/ml Ctx). Reference strains *Staphylococcus aureus* ATCC^®^29213, *Enterococcus faecalis* ATCC^®^29212, *E. coli* ATCC^®^25922, and *Pseudomonas aeruginosa* ATCC^®^27853 were examined in parallel as controls.

### Persistence of AR

AR stability in resistant isolates was determined according to the published procedures (Li et al., 2011) with slight modifications. Basically, after consecutive transfer every 12 h for 30 days, the cultures were serially diluted and plated on BHI agar plates. One hundred colonies were randomly picked from each sample, and spotted onto BHI agar plates with and without the corresponding antibiotic. The ratio of resistant to total colonies was used to describe the resistance persistence in ART isolates at the absence of the antibiotic selective pressure.

### Transformation

Chemical transformation was conducted using plasmids from nine Gram-negative ART isolates carrying AR genes and *E. coli* DH5α as a recipient by the calcium chloride transformation method (Dagert and Ehrlich, 1979). Meanwhile, electroporation was employed when introducing plasmids from five Gram-positive ART isolates carrying AR genes to *Lactococcus lactis* LM 2301 following the procedures described previously (Mcintyre and Harlander, 1989).

## Results

### MIC Assessment

MIC analysis showed that the MIC results of Tet, Erm, or Ctx for the four reference strains were comparable in BHI and CAMH broth. However, culture medium had varied but clear impact on MIC results of Sul/Tri. For instance, while the MIC values of Sul/Tri for *P. aeruginosa* ATCC^®^27853 in both media were comparable (608/32 μg/ml), the other three reference strains showed 4- to 64-fold higher MIC values in BHI than that of in CAMH. It is worth noting that *E. faecalis* ATCC^®^29212 didn’t grow as well in CAMH as in BHI broth.

As illustrated in **Table 2**, twenty-nine isolates used in the MIC assessment were identified and they belonged to 15 genera. MIC values for Tet, Erm, or Ctx in BHI were the same as, or twofold to fourfold higher than the results from CAMH. Among the strains examined, 9 out of 20 (45%) Ter^r^, 6 out of 6 (100%) Erm^r^, and 2 out of 5 (40%) Ctx^r^ isolates exhibited high and consistent MIC values (no less than 128 μg/ml) in both BHI and CAMH for Tet, Erm, or Ctx, respectively. But the MIC values for Sul/Tri in BHI and CAMH varied significantly in close to 70% of the isolates (21 out of 29). Some Sul/Tri^r^ isolates showed very high value (no less than 608/32 μg/ml) in both BHI and CAMH, including *Plesiomonas* sp., *Aeromonas* sp., and *Psychrobacter* sp. Bacteria from genera such as *Enterobacter* sp., *Bacillus* sp., and *Kurthia* sp. showed higher MIC value in BHI (no less than 304/16 μg/ml) than that in CAMH (no more than 9.5/0.5 μg/ml). Moreover, isolates of *Vagococcus* sp.*, Aerococcus* sp., *Corynebacterium* sp., and *Enterococcus* sp. didn’t grow or had poor growth in CAMH, but exhibited higher MIC value in BHI (no less than 608/32 μg/ml).

**Table 2:** Comparison of MIC results between BHI and CAMH medium.

Isolates	Identity	AR genes carried	Resistance phenotype and MIC (μg/ml)					

			**Sul/Tri^r^**		**Tet^r^**		**Erm^r^**		**Ctx^r^**	
			**BHI**	**CAMH**	**BHI**	**CAMH**	**BHI**	**CAMH**	**BHI**	**CAMH**
17iT21	*Plesiomonas* sp.	*sul*1	>608/32	>608/32	256	128	*–*	*–*	–	–
17wT9	*Plesiomonas* sp.	*tet*M	–	–	256	128	*–*	*–*	–	–
18iS4	*Plesiomonas* sp.	*sul*2	>608/32	>608/32	–	–	*–*	*–*	–	–
18iS31	*Plesiomonas* sp.	*sul*2	>608/32	>608/32	–	–	*–*	*–*	–	–
17fS1	*Enterococcus* sp.	*tet*M, *tet*L	>608/32	Weak growth ≤9.5/0.5	256	128	*–*	*–*	–	–
17fS3	*Enterococcus* sp.	*sul*1	>608/32	Weak growth ≤9.5/0.5	16	16	*–*	*–*	–	–
18iX15	*Enterococcus* sp.	*tet*S, *tet*L	>608/32	Weak growth ≤9.5/0.5	256	128	*–*	*–*	512	512
18fT19	*Enterococcus* sp.	*tet*S	>608/32	Weak growth ≤9.5/0.5	256	128	*–*	*–*	–	–
17iE3	*Enterobacter* sp.	*sul*2	304/16	≤9.5/0.5	–	–	512	256	–	–
17iE10	*Enterobacter* sp.	*sul*2	>608/32	≤9.5/0.5	*–*	*–*	512	256	*–*	*–*
17iE11	*Enterobacter* sp.	*sul*2	304/16	≤9.5/0.5	*–*	*–*	512	256	16	8
17iE26	*Enterobacter* sp.	*sul*2	>608/32	≤9.5/0.5	*–*	*–*	512	256	*–*	*–*
17fT6	*Kocuria* sp.	*sul*1	>608/32	≤9.5/0.5	64	64	*–*	*–*	*–*	*–*
17fT2	*Corynebacterium* sp.	*sul*1	>608/32	Weak growth	256	128	*–*	*–*	*–*	*–*
18fT26	*Corynebacterium* sp.	*tet*L	*–*	*–*	32	32	*–*	*–*	*–*	*–*
18fT27	*Corynebacterium* sp.	*tet*L	*–*	*–*	32	32	*–*	*–*	*–*	*–*
18iS7	*Aeromonas* sp.	*sul*1	>608/32	>608/32	*–*	*–*	*–*	*–*	*–*	*–*
18fE1	*Bacillus* sp.	*erm*B	>608/32	≤9.5/0.5	256	256	512	512	4	4
17fT12	*Aerococcus* sp.	*tet*M	>608/32	Weak growth	256	128	*–*	*–*	*–*	*–*
17fS5	*Aerococcus* sp.	*tet*M	>608/32	Weak growth	32	32	*–*	*–*	*–*	*–*
18fS13	*Psychrobacter* sp.	*sul*2	>608/32	>608/32	32	32	*–*	*–*	*–*	*–*
18fS19	*Psychrobacter* sp.	*sul*1, *sul*2	>608/32	>608/32	32	32	*–*	*–*	*–*	*–*
18pwE5	*Staphylococcus*sp.	*erm*C	>608/32	≤9.5/0.5	*–*	*–*	>512	>512	>512	>512
18fT47	*Carnobacterium* sp.	*tet*S, *tet*L	*–*	*–*	64	64	*–*	*–*	*–*	*–*
18fT5	*Kurthia* sp.	*tet*M	>608/32	≤9.5/0.5	256	256	*–*	*–*	64	16
18fT29	*Lactobacillus* sp.	*tet*L	*–*	*–*	32	32	*–*	*–*	*–*	*–*
18fS10	*Pseudoclavibacter* sp.	*sul*1	608/32	608/32	32	32	-	*–*	*–*	*–*
18fT12	*Vagococcus* sp.	*tet*L	>608/32	No growth	128	No growth	*–*	*–*	*–*	*–*

*BHI, Brain heart infusion**;** CAMH, cation-adjusted Mueller Hinton.*

### Phenotypic-Resistant Profiles of the ART Isolates

Of the recovered Sul/Tri^r^, Tet^r^, Erm^r^, and Ctx^r^ isolates (from BHI plates) associated with fish intestine, surface rinsing water, fish feed, pond mud, and pond water samples, 3772 of 4767 total isolates (79.1%) showed resistance to more than one antibiotic, and 824 isolates (17.4%) were resistant to all four antibiotics tested. As shown in **Table 3**, multi-drug-resistant bacteria were common in all samples.

**Table 3:** Multi-drug-resistant isolates from aquaculture samples.

Sample	Isolates resistant to antibiotic			
	1AR^a^	2AR	3AR	4AR
Surface rinsing water (*n* = 850)	18.7% (159/850)	35.6% (303/850)	32.2% (274/850)	13.4% (114/850)
Fish intestine (*n* = 1045)	19.8% (207/1045)	58.5% (611/1045)	12.1% (126/1045)	9.7% (101/1045)
Pond mud (*n* = 1293)	13.4% (173/1293)	34.9% (451/1293)	34.0% (440/1293)	17.7% (229/1293)
Pond water (*n* = 1429)	8.7% (125/1429)	15.2% (217/1429)	50.6% (723/1429)	25.5% (364/1429)
Fish feed (*n* = 150)	36.0% (54/150)	31.3% (47/150)	22.0% (33/150)	10.7% (16/150)

*^a^Number of antibiotic(s) the isolates were resistant to.*

### Prevalence of the AR Genes and Identification of the Isolates

As shown in **Table 4**, more AR genes were detected in isolates associated with fish feed and fish intestine than surface rinsing water, pond mud, and pond water samples. For instance, a high percentage and broad spectrum of AR genes were found in isolates from fish feed samples, including *sul*1 (10.2%), *sul*2 (2.0%), *tet*L (13.3%), *tet*M (9.2%), *tet*S (6.1%), and *erm*B (1.0%). Within the 13 AR determinants examined, *tet*M had the highest detection rate (8.1%) in the isolates.

**Table 4:** The identity of AR gene carriers.

Sample (# isolates tested)	Detected AR genes (# positive isolates)	Gene carrier [AR gene (#isolates identified)]
Surface rinsing water (209)	*sul*1 (8), *sul*2 (1), *tet*M (30), *sul*1+*tet*M (5)	*Plesiomonas* sp. [*sul*1 (3), *sul*2 (1), *tet*M(3), *sul1*+*tet*M (2)]
Fish intestine (357)	*sul*1 (7), *sul*2 (15), *tet*L (1)*, tet*M (9), *sul*1+*tet*M (1), *tet*L+ *tet*S(1),	*Aeromonas* sp. [*sul*1 (1)]; *Enterobacter* sp., [*sul*2 (7)]; *Enterococcus* sp. [*tet*L (1), *tet*L+ *tet*S (1)]; *Plesiomonas* sp. [*sul*1 (3), *sul*2 (6), *sul*1+ *tet*M (1)];
Pond Mud (269)	*sul*1 (3), *sul*2 (1)	*Aeromonas* sp. [*sul*1 (1)]; *Plesiomonas* sp. [*sul*1 (1)]
Pond water (244)	*sul*1 (5), *tet*S (1), *tet*L+*tet*S (3), *erm*C(1)	*Exiguobacterium* sp. [*sul*1 (2)]; *Staphylococcus* sp. [*erm*C (1)]; *Lactococcus* sp. [*tet*S (1), *tet*L+*tet*S (2)];
Fish feed (98)	*sul*1 (9), sul2 (1), *sul*1+*sul*2 (1), *tet*L (4), *tet*M (2), *tet*S (3), *tet*L+ *tet*M (6), *tet*L+ *tet*S (2), *tet*L+*tet*M+*tet*S (1), *erm*B (1)	*Aerococcus* sp. [*tet*M (2)]; *Bacillus* sp. [*tet*S (1), *erm*B (1)]; *Carnobacterium* sp. [*tet*L+*tet*S (1)]; *Corynebacterium* sp. [*sul*1 (1), *tet*L (2)]; *Kurthia* sp. [*tet*M (1)]; *Enterococcus* sp. [*sul*1 (1), *tet*S (2), *tet*L+*tet*M (5), *tet*L+*tet*S (1), *tet*L+*tet*M+*tet*S (1)]; *Kocuria* sp. [*sul*1 (4)]; *Lactobacillus* sp. [*tet*L (1)]; *Pseudoclavibacter* sp. [*sul*1 (1)]; *Psychrobacter* sp. [*sul*2 (1), *sul*1+*sul*2 (1)]; *Staphylococcus* sp. [*sul*1 (1)]; *Vagococcus* sp. [*tet*L (1)]

As illustrated in **Table 4**, identified AR gene carriers belonged to 18 genera. *Aeromonas* sp., *Enterococcus* sp., *Enterobacter* sp., and *Plesiomonas* sp. identified in fish intestine samples were commonly isolated from fish (Austin, 2002). Some of the isolates examined, including *Bacillus* sp., *Carnobacterium* sp., *Corynebacterium* sp., *Enterococcus* sp., *Plesiomonas* sp.*, Lactococcus* sp., and *Psychrobacter* sp. were found to carry multiple resistance encoding genes. Fish feed contained various AR genes in a broad spectrum of organisms, though total count of ART bacteria was relatively low.

### Stability of the AR

Results of persistence assessment showed that 90–100% of the progenies from nine ART isolates examined retained their original AR traits, indicating that the AR determinants are generally stable in the resistant isolates without the corresponding antibiotic selective pressure. Progenies of an *Enterococcus* strain from the intestine sample retained resistance to Sur/Tri but became susceptible to Tet and Ctx.

### Functionality of the AR Determinants

Plasmids from two out of nine Gram-negative isolates (*sul*1^+^ *Aeromonas* sp. from the fish intestine and *sul*2^+^ *Psychrobacter* sp. from the fish feed) and one out of five Gram-positive isolate (*tet*L^+^ *Vagococcus* sp. from fish feed) were successfully transferred to *E. coli* and *L. lactis*, respectively, resulting in acquired resistance in transformants. The transformants exhibited comparable MIC for the corresponding antibiotic with the donors, indicating the resistance genes were functional in other bacteria if acquired via horizontal gene transfer events.

## Discussion

It is recognized that AR ecology, of the emergence, persistence, and dissemination of ART bacteria and AR genes in the microbial ecosystem, is much more complicated than previously thought. Up to 10^7^ CFU/g ART bacteria, representing about 1% of the total bacterial population, were detected in the samples from this aquaculture farm, despite that the farm has no known history of antibiotic applications during production (Huang, 2014). Here, we report that majority of the ART isolates from the fish and aquaculture environment were resistant to two or more antibiotics.

MIC is a relatively precise measure of resistance to antibiotics. The measurement is essential for AR assessment not just because of the need to measure resistance trend, but more importantly, the MIC value is correlated to the dosage required for effective therapy. It is important to recognize that bacterial strains carrying the same resistance gene may have different MIC values, even for those from the same genus (Miranda et al., 2003). Understanding the molecular mechanisms contributing to the phenotype would be of great value to properly evaluate AR risk and control its dissemination. However, it has been difficult to compare antibiotic susceptibility results because of variation in testing methods in published studies. Most studies in the United States used CAMH broth, following NCCLS-recommended procedure (now it is CLSI), while Iso-Sensitest broth is widely used in Europe (Koeth et al., 2000). Koeth et al. (2000) conducted a study using 124 Centers for Disease Control and Prevention (CDC) reference strains compared the medium effect on MIC results, and concluded that data from the above two broth were comparable.

Due to genetic diversity of microbiota associated with both host and natural environment, bacterial media used in the studies likely have an impact on the results. Sul/Tri^r^ population was found abundant in microbiota across the host and environmental samples. Considering the potential effect of cultural medium on MIC results, in this study we have examined MIC for representative strains in both CAMH and BHI. The isolates had different MIC values in the two media, especially for Sul/Tri^r^ isolates. Approximately 67% Sul/Tri^r^ isolates recovered from BHI medium exhibited resistance against Sul/Tri in CAMH medium (data no shown), and some of them had much higher MIC values in BHI than that in CAMH. As shown in **Table 2**, some isolates such as *Enterococcus* sp. (17fS1, 17fS3, 18iX15, and 18fT19) and *Enterobacter* sp. (17iE3, 17iE10, 17iE11, and 17iE26) were classified as susceptible to Sul/Tri using CAMH, but showed high MIC values in BHI. In fact, the corresponding resistance determinants *sul*1 or *sul*2 were identified in the above strains with positive detection rate comparable to other AR genes, suggesting their MIC results in CAMH-Sul/Tri broth cannot represent their real resistance status against the antibiotics. As a matter of fact, CLSI has acknowledged the mismatch between MIC results of *Enterococcus* sp. using CAMH-Sul/Tri medium and resistant patterns in clinical treatments (CLSI, 2013). Moreover, although CAMH medium is widely used for MIC test, bacteria from certain genera didn’t grow well in CAMH. As shown in **Table 2**, one *tet*L bearing isolate *Vagococcus* sp. could not grow in CAMH-Tet, but showed high MIC value (128 μg/ml) in BHI-Tet. These data indicated the limitations of CAMH as the medium for MIC assessment. While we have found that BHI serves the need for MIC analysis for most of the commensal isolates in this study, further validation using more reference strains and supplementation of media for cultures discriminated by BHI will be essential for methodology improvement. Moreover, ART isolates with higher MIC values are more difficult to eliminate with antibiotics. Whether the high MICs of the above strains are due to new molecular determinants or established resistance genes but with additional enhancement mechanisms are worth further investigation.

As illustrated in **Table 4**, 30 of 98 ART isolates (30.6%) from fish feed samples were found to carry one or more of the 12 AR genes examined, and the positive detection rate was much higher than those of the ART isolates from other samples, being 9.5% (34 of 357) in fish intestine, 21% (44 of 209) in surface rinsing water associated with skin microbiota, 4.1% (10 of 244) in pond water, and 1.5% (4 of 269) in pond mud sample. This result was consistent with the finding that multiple AR gene pools in high levels were detected in fish feed samples by real-time PCR (Huang, 2014). In addition, fish skin and intestine samples also contained a large number of ART bacteria and AR genes which also is in agreement with the results reported by Ye et al. (2013). Because animal and fish by-products, often rich in ART bacteria, are used in fish feed as an important protein source, it is inevitable that there is a large pool of AR genes and potentially AR gene carrying bacteria in fish feed. ART bacteria even multi-drug-resistant bacteria were reported in various types of animal feeds, such as poultry feeds (Schwalbe et al., 1999), cattle feed ingredients (Dargatz et al., 2005), and rendered animal protein products originating from poultry, cattle, and fish (Hofacre et al., 2001). Without proper treatment, fish feed rich in AR gene-carrying bacteria survived the feed manufacturing process could be a potential risk factor spreading ART bacteria in the aquaculture production system and subsequently the food chain. In fact, several genera of ART bacteria were found in multiple types of samples. For example, *Plesiomonas* sp. were detected in surface rinsing water, fish intestine, and pond mud samples, while *Aeromonas* sp. were present in fish intestine and pond mud samples. The data suggested that these microorganisms were present in both the host and farm environment, and may have been circulating within the aquaculture ecosystem.

As illustrated in **Table 4**, there was no significant correlation of the types of AR encoding genes and their carriers between fish intestine and feed, indicating that hosts (fish) potentially also have a role in the selective enrichment of or breeding certain resistant bacteria in the population. In this study, the *sul*1 gene of *Aeromonas* sp. from the fish intestine, the *sul*2 gene of *Psychrobacter* sp. and the *tet* (L) gene of *Vagococcus* sp. from the fish feed were found functional after being transferred to the corresponding recipients, suggesting that they can serve as a source of AR genes if involved in horizontal gene transfer events. Finally, representative AR genes were only found in a small percent of the ART isolates. A functional genomic analysis has already discovered a new Tet^r^-encoding gene, *tet*47, from a Tet^r^ isolate associated with a fish intestine sample (Huang et al., 2015).

In summary, ART commensal bacteria associated with this aquaculture system with no known history of antibiotics application exhibited multi-drug-resistance (MDR). Various AR determinants were detected and 18 bacterial genera were identified among those AR gene carrying isolates. The data suggested that the aquaculture system is a rich reservoir of AR, risk factors other than direct antibiotic application, such as AR-rich feed or even environmental factors, may have played important role(s) disseminating AR in this ecosystem. Follow-up studies are needed to reveal a more comprehensive picture of AR in aquaculture production, and for targeted and effective AR mitigation in the ecosystem. Moreover, BHI medium was found more suitable for majority of the commensal bacteria examined than CAMH broth for MIC assessment.

## Conflict of Interest Statement

The authors declare that the research was conducted in the absence of any commercial or financial relationships that could be construed as a potential conflict of interest.
